# Influence of female cuticular hydrocarbon (CHC) profile on male courtship behavior in two hybridizing field crickets *Gryllus firmus* and *Gryllus pennsylvanicus*

**DOI:** 10.1186/s12862-020-1587-9

**Published:** 2020-02-04

**Authors:** Brianna Heggeseth, Danielle Sim, Laura Partida, Luana S. Maroja

**Affiliations:** 1 Department of Mathematics and Statistics, Williams College, Williamstown, MA USA; 2 Department of Mathematics, Statistics, and Computer Science, Macalester College, St. Paul, MN USA; 3 Department of Biology, Williams College, Williamstown, MA USA

**Keywords:** Mate choice, Hybrid zone, Pre-mating barrier, Speciation, Introgression

## Abstract

**Background:**

The hybridizing field crickets, *Gryllus firmus* and *Gryllus pennsylvanicus* have several barriers that prevent gene flow between species. The behavioral pre-zygotic mating barrier, where males court conspecifics more intensely than heterospecifics, is important because by acting earlier in the life cycle it has the potential to prevent a larger fraction of hybridization. The mechanism behind such male mate preference is unknown. Here we investigate if the female cuticular hydrocarbon (CHC) profile could be the signal behind male courtship.

**Results:**

While males of the two species display nearly identical CHC profiles, females have different, albeit overlapping profiles and some females (between 15 and 45%) of both species display a male-like profile distinct from profiles of typical females. We classified CHC females profile into three categories: *G. firmus*-like (F; including mainly *G. firmus* females), *G. pennsylvanicus*-like (P; including mainly *G. pennsylvanicus* females), and male-like (ML; including females of both species). *Gryllus firmus* males courted ML and F females more often and faster than they courted P females (*p* < 0.05). *Gryllus pennsylvanicus* males were slower to court than *G. firmus* males, but courted ML females more often (*p* < 0.05) than their own conspecific P females (no difference between P and F). Both males courted heterospecific ML females more often than other heterospecific females (*p* < 0.05, significant only for *G. firmus* males).

**Conclusions:**

Our results suggest that male mate preference is at least partially informed by female CHC profile and that ML females elicit high courtship behavior in both species. Since ML females exist in both species and are preferred over other heterospecific females, it is likely that this female type is responsible for most hybrid offspring production.

## Background

To fully understand mate choice and its influence on speciation, we need to understand the mechanisms behind this choice. Mate choice in the form of preference for conspecifics is a pre-zygotic barrier that prevents gene flow between species and is important because by acting early in the life cycle, it can prevent more gene flow than other later acting barriers [10]. While mate choice has traditionally been almost synonymous with female mate choice [28], male mate choice, or preference, has now been reported even in species with little male parental care [15]. Males exhibit differential courtship behavior to females based on various traits such as size [4, 16, 22], relatedness [6, 35, 56] and species membership [25, 38, 44].

The morphologically and behaviorally similar hybridizing field crickets, *Gryllus firmus* [53] and *Gryllus pennsylvanicus* [19], provide an opportunity to better understand the role of male mate preferences in reproductive isolation. These two species form a well-described mosaic hybrid zone [20, 30, 48] and have several barriers that limit gene exchange [14, 19, 31, 37]. Their pre-mating barrier is largely explained by differential male courtship; males court conspecific females more readily and intensely than they court heterospecifics [38]. Therefore, while female crickets ultimately decide whether or not to mate (as they have to mount the male), male courtship intensity plays a significant role in their decision and females often mate with intensely courting males and will never mate a non-courting male [38].

While barriers to gene exchange are well described in these crickets, the mechanism behind this male mate preference is not understood. While morphological differences are often used in mate recognition, this is unlike to be the case between the morphologically similar *G. firmus* and *G. pennsylvanicus* which might instead use chemical signals such as cuticular hydrocarbons (CHCs). These compounds serve as contact pheromones to a wide variety of insects [2, 11, 12, 17, 33, 34, 50], are sexually dimorphic in many species [8, 9, 24, 59], including field crickets [38, 42, 61–64, 67], and are used for mate choice in various insect species [23, 45, 54, 59, 60]. In *G. firmus* and *G. pennsylvanicus* CHC composition is different between sexes however, while males of both species share the same composition, females of the two species are different but overlapping [38]. Furthermore, a subset of females from both species (between 15 and 45% based on this and previous data) exhibits a pattern that is typical of that of a male (male-like females, ML), the relevance of this pattern is unknown. This female CHC profile variability, with both unique and shared patterns between species, suggests that it could be the mechanism behind male mate recognition and could thus explain why males sometimes, but not always, court heterospecifics.

Our goal is to test the hypothesis that female CHC profile informs male mate preferences in the hybridizing field crickets *G. firmus* and *G. pennsylvanicus*. We show that males of both species mate with heterospecific ML (male-like) females at a higher rate than other heterospecifics, suggesting that males can indeed detect and use CHC information for courtship decisions. Therefore, it is possible that ML (male-like) females produce most of the hybrid offspring in the hybrid zone.

## Results

### Cuticular hydrocarbon analysis

For the gas chromatography analysis, we used the similar methods of Maroja et al. [38], we scored 17 peaks (Table 1) in 259 individuals (GP♂: *n* = 67, GP♀: *n* = 65, GF♂: *n* = 68, GF♀: *n* = 59). Males typically have fewer peaks than females with less variation between individuals (Table 1). Size (GP♂ 5.67 ± 0.47 cm, GP♀:5.90 ± 0.30 cm, GF♂: 5.42 ± 0.42 cm, GF♀: 5.76 ± 0.34 cm) was significantly different between sexes (F_1, 249_ = 35.0, *p* < 0.001) and between species (F_1, 249_ = 16.6, p < 0.001), but there is no significant interaction between sex and species (F_1, 249_ = 1.4, *p* = 0.24) in a two-way ANOVA, however, unlike previous studies, *G. pennsylvanicus* were the larger species in our sample [30].

**Table 1:** Average relative proportion and standard deviation of the 17 scored peaks for CHC analysis

PEAKS	GP ♂ (*n* = 67)			GP ♀ (*n* = 65)			GF ♂(*n* = 68)			GF ♀ (*n* = 59)		
	Mean	SD	% zero	Mean	SD	% zero	Mean	SD	% zero	Mean	SD	% zero
F0	0.00	0.00	100.00	10.28	12.61	8.96	0.00	0.00	100.00	0.17	0.38	77.97
F4	0.02	0.06	89.55	1.16	1.16	14.93	0.24	0.42	48.53	1.47	1.40	22.03
M0	2.78	1.37	2.99	17.36	13.22	1.49	2.38	1.38	0.00	4.62	3.79	10.17
F5	0.00	0.00	100.00	7.84	9.55	23.88	0.00	0.00	100.00	2.84	3.54	25.42
M1	8.53	4.10	2.99	7.69	4.66	0.00	8.33	5.17	0.00	6.79	3.68	1.69
F8	1.37	0.85	4.48	3.37	2.94	1.49	1.10	0.73	4.41	3.02	1.89	5.08
M3	4.25	3.36	14.93	2.77	2.04	13.43	6.48	4.18	14.71	5.03	2.82	3.39
M4	17.81	17.07	38.81	14.30	14.98	31.34	24.01	15.63	19.12	6.44	9.96	37.29
M5	33.86	16.50	10.45	10.65	11.06	4.48	25.87	12.26	7.35	6.93	8.89	16.95
M6	16.66	9.26	7.46	10.85	4.14	0.00	15.38	7.35	1.47	19.12	7.73	3.39
F10	0.02	0.08	92.54	3.44	4.15	16.42	0.04	0.14	91.18	8.93	6.46	3.39
M7	8.03	8.12	38.81	4.42	4.56	34.33	9.08	6.49	22.06	11.37	5.31	1.69
F11	0.00	0.00	100.00	0.20	0.59	70.15	0.03	0.09	91.18	1.21	0.93	20.34
F12	0.35	1.95	74.63	0.85	1.37	35.82	1.55	1.48	13.24	8.18	9.22	3.39
M8	6.28	8.14	38.81	3.20	4.49	40.30	5.28	5.76	42.65	2.61	3.64	49.15
F13	0.04	0.26	97.01	1.47	3.51	44.78	0.01	0.07	95.59	6.73	6.30	18.64
F14	0.00	0.00	100.00	0.16	0.49	85.07	0.22	0.81	86.76	4.54	3.52	10.17

**Fig. 1:**
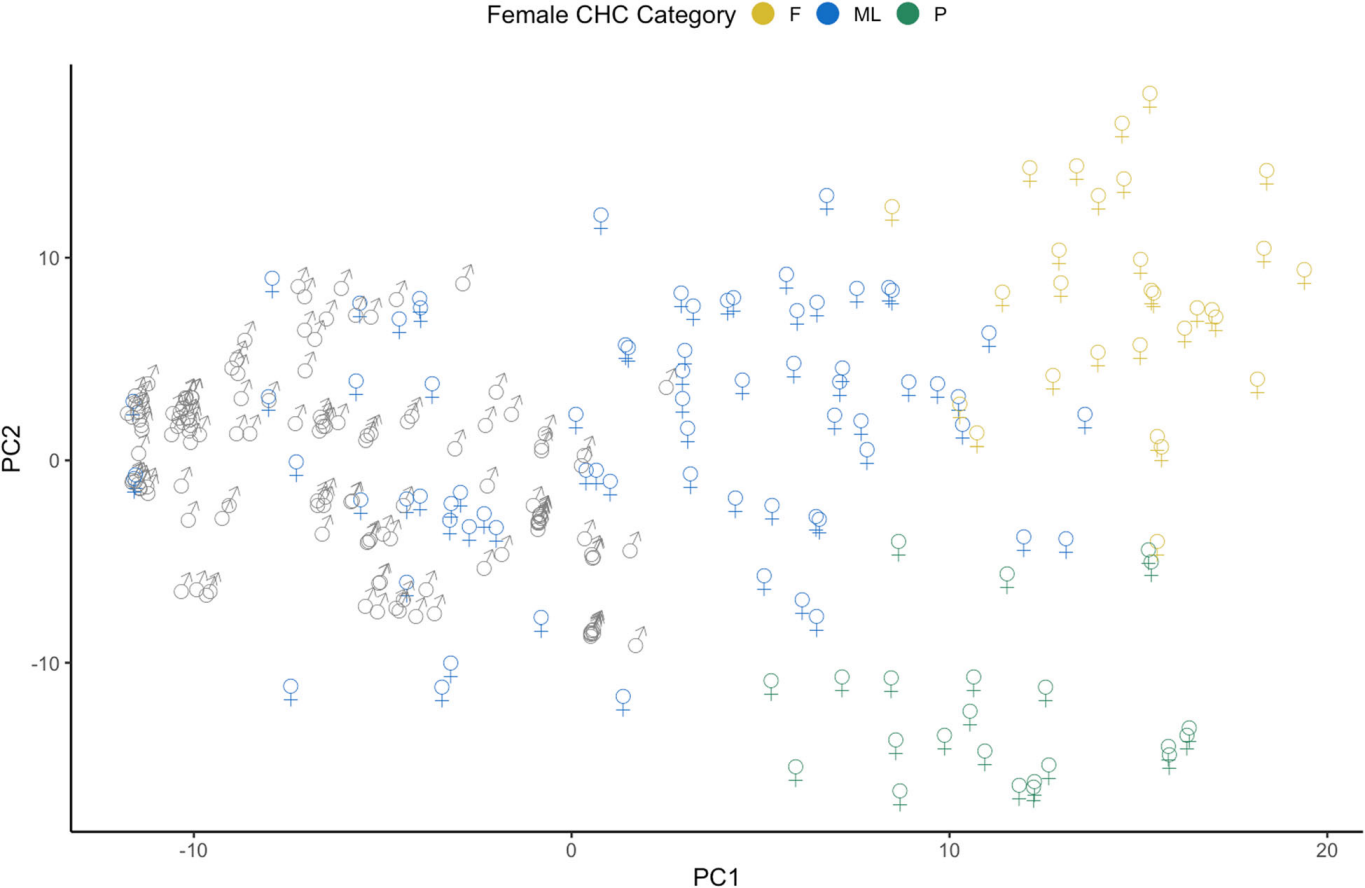
Clustering of CHC for females. Principal components for relative peak proportion for all crickets, labeled by sex and female CHC categories generated through clustering analysis. Categories include cluster “F” (predominantly *G. firmus* females, *n* = 29), cluster “P” (only *G. pennsylvanicus* females, *n* = 23), and cluster “ML” (females with male-like CHC profiles, *n* = 32 for *G. firmus* and *n* = 40 for *G. pennsylvanicus*)

As reported before [38], the first two principal components of relative CHC peak proportion (percent of each peak) and composition (presence or absence of peak) were less varied within males of both species with the area of the convex hull for males equal to 4.8 and 25.3 as compared to 46.6 and 38.8 for females, for peak proportion and composition respectively (Fig. 1). Moreover, while females had significantly different CHC profiles between species [38], some of the profiles were overlapping between species and some females exhibited male-like profiles (Figure 4 in Appendix and Fig. 1). Male or female size were not associated with CHC profile and therefore were not included in the analyses.

Using the results from a cluster analysis of female CHC profile, we classified females into three distinct CHC categories (Fig. 1): “F” (*G. firmus* females, all but 2 individuals in this category are *G. firmus*, *n* = 29), “P” (*G. pennsylvanicus* only, all individuals in this category are *G. pennsylvanicus*, *n* = 23), and “ML” (females that have male-like CHC profiles – both species are in this category, *n* = 32 for *G. firmus* and *n* = 40 for *G. pennsylvanicus*). Since these two of the clusters correlate well with female species, we focus on the third cluster of females that have male-like profiles and consider female species in further analyses.

### Courtship success and female profile types

**Fig. 2:**
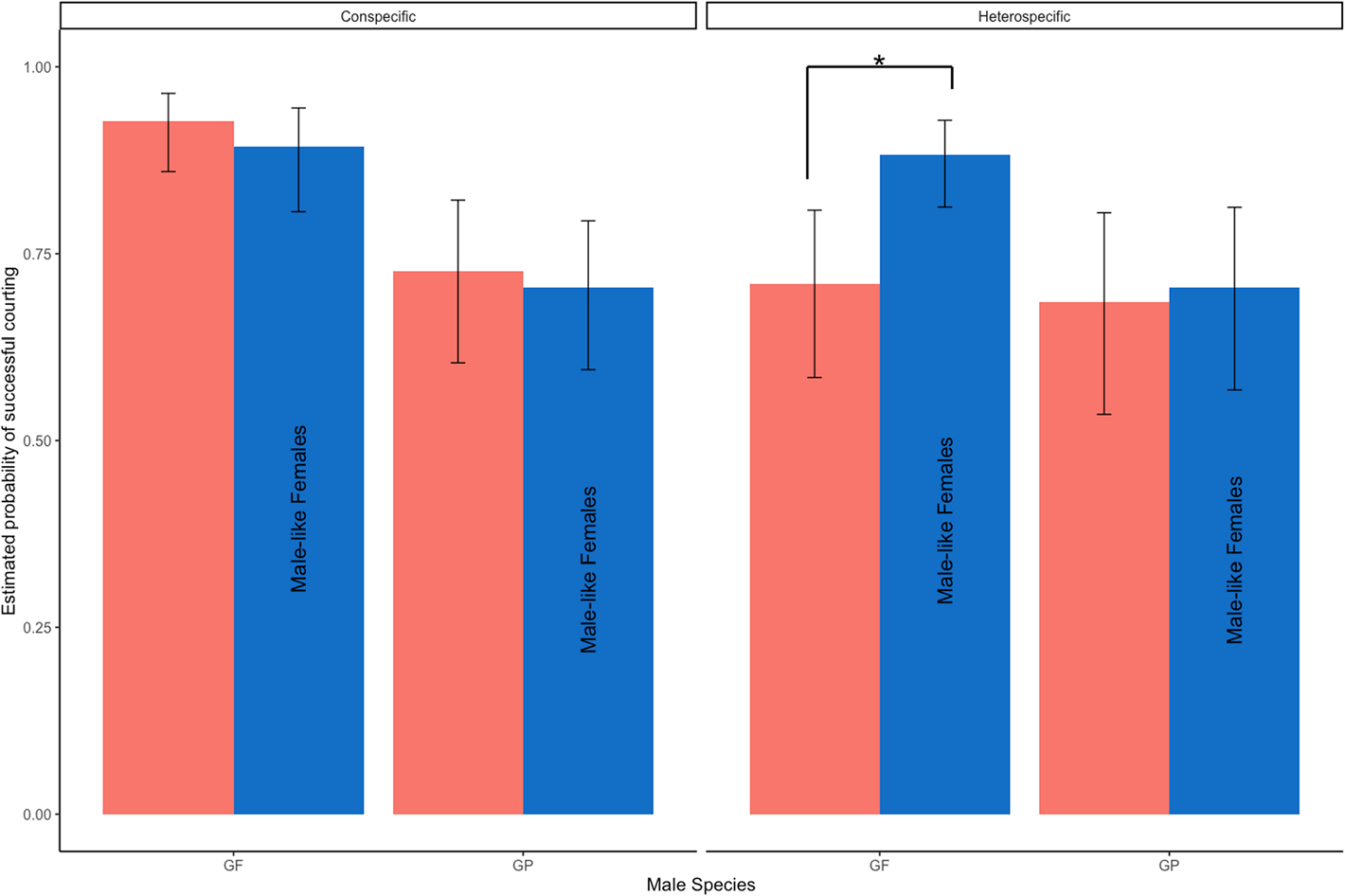
Courtship success by male species, crossing type and female CHC category. Estimated proportion of successful courtships by male species, crossing type (conspecific or heterospecific) and male-like female CHC category (ML, blue) or not (red) from mixed effects binomial logistic regression model with 95% confidence intervals

Among pairings with *G. firmus* males, the proportion of courtship success was greatest with conspecific females (85%), followed by male-like (ML) heterospecific females (79%) and then the lowest success rate (66%) was with heterospecific females that do not have a male-like CHC profile (Fig. 2). The highest rate of courtship initiation with *G. pennsylvanicus* males happened with heterospecific ML females (68%) and conspecific females (66%).

A binomial logistic mixed effects model was fitted to the data to test whether the courtship success of *G. firmus* or *G. pennsylvanicus* males was impacted by female species and CHC categories while accounting for the baseline variation between individual crickets. Figure 2 shows estimated probabilities of courtship success with associated 95% confidence intervals. In general, among conspecific pairs, courtship success is similar between CHC profiles (GF: *p* = 0.31, GP: *p* = 0.68), but in heterospecific pairings, success was more likely for male-like CHC profiles (GF: *p* < 0.001, GP: *p* = 0.80) (Fig. 2). Table 2 gives the estimated odds ratios of courtship success between male-like and not male-like CHC profile for each male species and type of pair crossing. For conspecific pairings, we estimated that courtship was less likely for male-like females than those with species-specific CHC profiles, but we do not have enough evidence to claim a statistical discernable difference. For heterospecific pairings, the odds of male *G. firmus* crickets to engage in courting behavior with a female bearing the male-like profile were 2.58 times more that of a female with a species-specific profile (95% CI: 1.314, 5.079). For male *G. pennsylvanicus* crickets, we estimate the odds of courtship success with a female bearing a male-like profile was 1.1 times that of a female with a species-specific CHC profile, but we do not have the power to claim a statistical discernable difference (95% CI: 0.604, 2.247).

**Table 2:** Analysis of Courtship Success and Time to Courtship by Male Species, Crossing Type, and Male-like Female CHC Category

	Female CHC Category	Courtship Success Odds Ratio (95% CI)	Courtship Time Hazard Ratio (95% CI)
Conspecific Pairs			
Male Species: GF	ML v. Not ML	0.781 (0.337, 1.673)	1.176 (0.858, 1.613)
Male Species: GP	ML v. Not ML	0.957 (0.496, 1.846)	0.910 (0.645, 1.283)
Heterospecific Pairs			
Male Species: GF	ML v. Not ML	2.583 (1.314, 5.079)	1.610 (1.166, 2.222)
Male Species: GP	ML v. Not ML	1.165 (0.604, 2.247)	1.039 (0.731, 1.478)
Random Intercepts	Binomial Logistic Mixed Effects Estimated SD	Cox Proportional Hazard Mixed Effects Estimated SD	
Male ID	1.435	0.714	

Binomial logistic mixed effects regression analysis of courtship success odds ratios and 95% confidence intervals and the estimated standard deviations of random intercepts are reported for males. Cox proportional hazard mixed effects hazard ratios and 95% confidence intervals and the estimated standard deviations of random intercepts for males. Hazard ratios are interpreted as the relative courtship rate at a time t of one group as compared to another. For example, at any point in the trial, *G. firmus* males are 1.610 more likely to start courting with a heterospecific females with a male-like (ML) CHC profile then females with any other CHC profile

### Time to initiate courtship

**Fig. 3:**
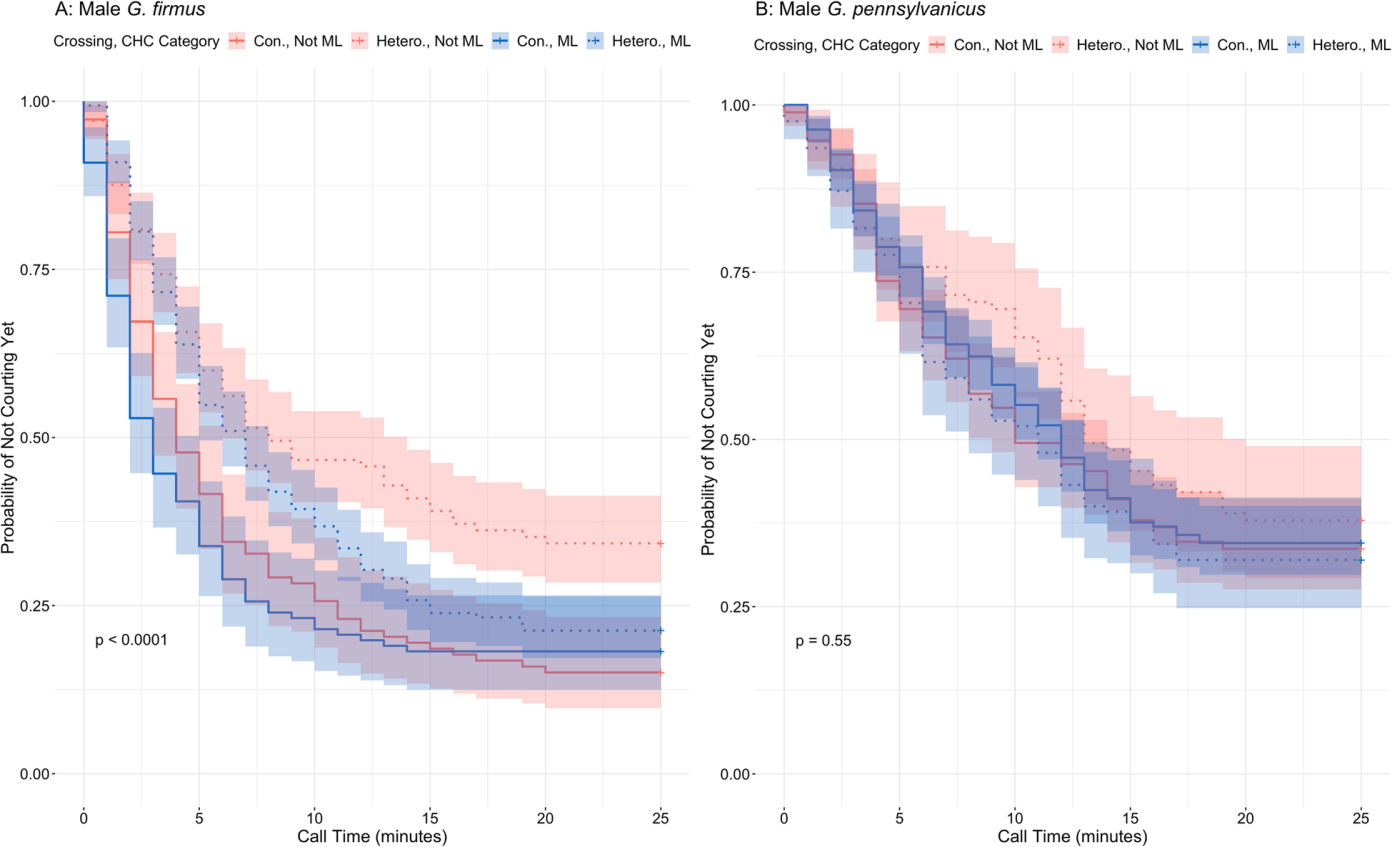
Kaplan Meier (KM) curve of time to courtship for *G. firmus* males (**a**) and for *G. pennsylvanicus* males (**b**) by crossing type (Con = conspecific; Hetero = heterospecific) and male-like female CHC category. The KM curve estimates the probability of not yet successful courting at a given time. The time to courtship differs significantly by crossing type and male-like CHC category for *G. firmus* males (*p* < 0.0001), but not for *G. pennsylvanicus* males

To analyze the time males took to court females, we measured how long males took to initiate the courtship call. A survival analysis was fitted to this call time data for both *G. firmus* and *G. pennsylvanicus* males to estimate the probability that a male has not yet courted at a given time for each female CHC category. Survival analysis models time to event data, and in this context, the event is successful courtship. For *G. firmus* males, conspecific females were courted significantly more quickly than heterospecific females (Fig. 3). We also note that *G. firmus* males were significantly more like to quickly court a male-like heterospecific female than another heterospecific female. For *G. pennsylvanicus* males, the estimated Kaplan-Meier curves are not significantly different across the female species and male-like CHC categories.

The Cox proportional hazards mixed effect models the hazard function, which is the instantaneous rate of successfully courtship at a given time, as a function of male species, type of pair crossing, male-like or not male-like CHC category, and all of their interactions. At any point in the trial, we estimated *G. firmus* males were 1.61 (95% CI: 1.166, 2.22) times more likely to start courting ML profile females than females with other profiles in heterospecific pairings and while not significant, the estimated chance of courtship initiation with ML profile females was 1.176 (95% CI: 0.858, 1.613) times that of other profile females in conspecific pairings (Table 2). No significant differences in the time to courtship events were detected among female CHC categories for *G. pennsylvanicus* males.

## Discussion

Our study suggests that female CHC profile can inform male mate preferences at least in one of the hybridizing field cricket species: *G. firmus*. *Gryllus firmus* males treated females with distinct CHC profiles differently and, even after controlling for female species (heterospecific vs. conspecific females), male *G. firmus* had a higher courtship success with heterospecific ML (male-like) than other heterospecific females. While differences were not significant for *G. pennsylvanicus* males, the direction of the preference was also towards ML females.

To our knowledge, this is the first report pointing to the importance of female CHC profile in male mate choice. In species that lack parental care, male mate choice is rare [15], but can evolve in species where females are found simultaneously [3] and/or courtship is expensive [5] or exposes males to predators [15]. Crickets fulfill these requirements; in our species, populations are locally abundant (especially for *G. firmus*) with dozens of individuals often living under a single rock or wooden plank (LSM personal observation). Furthermore, courtship can be costly, not only because the calling male exposes himself to predators and parasitoids, but also because the spermatophore is nutrient rich and often consumed by females. We need further data to fully understand the evolution of male mate choice. For instance, it is unclear if female CHC profile serves as an indicator of female fitness or if males are more able to detect certain chemicals. This information will be essential in understanding the selective basis behind the evolution of male mate choice in this system.

Here we confirmed previous results [38] showing that *G. firmus* and *G. pennsylvanicus* males exhibited similar CHC profiles and that females varied within and between species and had profiles that were distinct from that of males (although some females displayed a male-like profile). Sexual dimorphism of CHC profile is common and has been observed in several insect species such as burrowing bees [57], fireflies [41], and the Australian field cricket *Teleogryllus oceanicus* [60]. Importantly sexually dimorphic CHC profiles have been shown to mediate sex recognition in males, leading to aggression towards other males and courtship towards females [43, 66]. In closely related species, interspecific differences in CHC profile might be important as a reproductive barrier. Interspecific differences are known to exist in closely related *Drosophila* species (Etges & Ahrens, 2001 [7, 39, 54];) as well as between potentially hybridizing crickets such as the *Laupala* complex in Hawaii [42] and other field crickets [32, 65]. These interspecific differences in CHC profile have been implicated in influencing mate choice and therefore reproductive isolation.

### Implications to the hybrid zone and speciation

*Gryllus firmus* and *G. pennsylvanicus* have various prezygotic barriers in all life stages that reduce heterospecific matings [21, 37, 51]. Male mate preference seems to be a significant prezygotic behavioral barrier leading to a longer time or failure of courtship when the female is heterospecific [37, 38]. Furthermore, these crickets also have a unidirectional post-mating barrier to fertilization: only *G. pennsylvanicus* females can produce hybrid offspring [20, 29, 37]. It is thus important to understand the basis of male mate preference especially in *G. firmus* males, the only species capable of siring hybrid offspring.

To examine the influence of CHC composition on male mate preference, we measured courtship success as well as time to mate. We categorized females into species specific CHC compositions (*G. firmus* and *G. pennsylvanicus*) as well as a third category composed of females of both species which exhibit a CHC composition similar to that of males (male like; ML). When accounting for female species, male *G. firmus* courted heterospecific ML females more often than other heterospecific females (Fig. 2). While CHC category alone was not entirely responsible for *G. firmus* male mate preference, as conspecific ML females were courted more quickly than heterospecific ML females (Fig. 3), it still played a role in the male mate preference. In agreement to previous studies, we also found that *G. pennsylvanicus* males court less often and did not discriminate between conspecifics and heterospecifics [37]; however, they too were more successful at courting ML females but not significantly so (Fig. 2).

Given that courtship in this system is initiated by males and thus mediated by male mate preference and that a female will only mate a male that is intensely courting [38] this preference for ML females, the only CHC category common in both species, is relevant to hybridization. Male-like females are common in both species constituting between 15 and 45% of the female population (based on this and other population surveys). Since *G. firmus* males court ML heterospecific females more successfully than other heterospecifics, this suggests that most of the hybridization happens through this female type.

### Male-like females is not an strategy to evade courtship

Initially female male-like profiles were hypothesized as a strategy to evade male harassment [38]. In studies with other *Gryllus* crickets, males are known to aggressively compete for acquisition of females [13, 55], and in other species, such as damselflies, male mimicry is often used to evade male harassment [18, 47]. In polyandrous *Ischnura* damselflies, females exhibit three different color morphs, one of which resembles the male coloring and aids in male courtship evasion [52]. In our sample a significant fraction of the females had male-like CHCs (GF: 54.2%, GP: 61.5%), however these females seemed to be favored by males, who courted them more or as intensely as they courted other conspecifics. We thus refute the hypothesis of courtship evasion previously proposed. Further studies are needed to elucidate why such phenotype is maintained in these species.

## Conclusions

We showed that *G. firmus* males courted heterospecific females with a male-like profile more successfully than other types of heterospecific females and, since *G. firmus* is the only male capable to siring hybrid offspring, these ML females might be responsible for most of the hybridization. We also showed that conspecific ML females seem to be either preferred (*G. pennsylvanicus*) or not discriminated against (*G. firmus*) males, thus refuting the hypothesis that females with a male-like profile could be evading courtship.

## Methods

### Collection

In August 2013, we collected penultimate instar *G. firmus* crickets from Guilford, CT (41°.13′,-72°40′) and *G. pennsylvanicus* crickets from Ithaca, NY (42°25′,-76°.29′), allopatric pure species populations. Individuals were separated by sex and species, and raised at room temperature (25 °C) in plastic cages (33 × 20 × 13 cm, with a maximum of 12 individuals) with ad libitum food (a mixture of Purina Cat Chow®, LM Bonanza Rabbit Food®, and Fluker’s Cricket Feed®) and water.

### Courtship trials

To measure male courtship intensity as a function of speed, we placed males in a petri dish with either a heterospecific or conspecific female and recorded the time to the start of courtship. We conducted four sets of crosses each day (10 AM and 2 PM). In each cross set (am and pm), a male was paired to a conspecific female followed by a heterospecific female an hour later (or reverse, heterospecific then conspecific). In total each male was placed with four conspecific and four heterospecific females over a two day period (alternating the order of conspecific and heterospecific females). Females were also tested to eight males, but were kept virgin throughout the experiment. Based on previous work, [38] we limited time to a maximum of 25 min (95% of males either already initiated or will not initiate courtship past this time), if the male did not initiate courtship within that time, the trial was considered unsuccessful. Pairs were never allowed to mate; after the male initiated courtship the pair was immediately separated. All male and female crickets used in the experiment were of approximately 12 days old (within 1–4 days apart); the adult lifespan in captivity is 30 ± 8 days (personal observation). We measured pronotum size as a proxy for body size in both males and females. and then compared species with a two-way ANOVA analysis.

### Cuticular hydrocarbon analysis

We extracted CHC from all individuals used in the courtship experiment by placing whole crickets into glass vials containing 3 mL (females) or 2 mL (males) of HPLC grade hexane for 5–7 min. For the analysis we transferred the CHC samples into 2 mL clear glass surestop vials with 300 μL glass inserts and analyzed with Agilent Technologies (AT) 7890A GC system with an (AT) HP-5 ms (325 °C 30 m × 250 μm x .250 μm) column attached to an AT 5975C inert XL EI/CI MSD with triple-Axis Detector MS system, that obtains chromatograms and both electron and chemical ionization mass spectra. The GCMS method consisted of a 2 μL of each sample injected in a split mode with a split ratio of 100:1. The column was held at an initial temperature of 100 °C for 1 min followed by 15 °C/min increase to 180 °C, then a 3 °C/min increase to 260 °C, and finally a 1 °C/min increase to a final temperature of 280 °C held for 10 min.

For GCMS data analysis, we scored a total of 17 peaks for each individual. Ten of these peaks were previously used as representative of common compounds in males and females [38] and we also scored seven new peaks. To score the peaks as a relative proportion of the total, we took the percent area contributed by each of the scored peaks and scaled the scored peaks to add up to 100% for each individual. To account for the dependence in the relative proportions, we used the centered log ratio (CLR) transformation prior to further analyzing the relative proportions of the peaks [1].

**Fig. 4:**
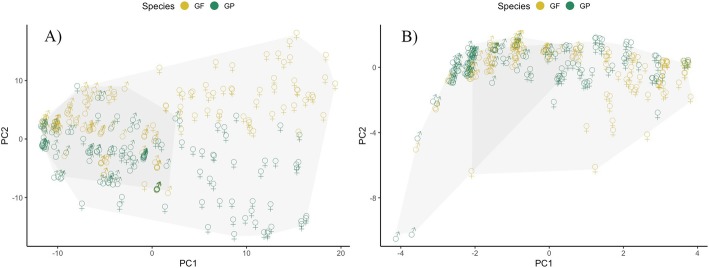
Principal component analysis of CHC for all individuals. **a** Relative peak proportion and **b** composition. Green represents *G. pennsylvanicus* and orange represents *G. firmus.* Sex is shown with male and female symbols

Cuticular hydrocarbon data was visualized via principal component analysis (Figure 4 in Appendix). Then the female CHC data were clustered into homogenous groups, or CHC categories, based on the Euclidean distance of CLR transformed relative proportions of the 17 peaks using the partitioning around medoids algorithm [27]. We chose the number of groups that maximized the average silhouette, a cluster validity measure which measures the cohesion and separation of the clusters [49]. This clustering process of female CHC profiles resulted in three clusters, two that correlated with species and a third that was the most similar to an average male CHC profile.

### Behavioral and CHC integration analysis

We used a binomial logistic mixed effects model to predict courtship success of each pair as a function of the species and female CHC profile category. Based on visualizations of courtship success rates, we included all main effects and pairwise interactions with male species, type of pair crossing (conspecific or heterospecific), and an indicator for “male-like” female CHC category and a three-way interaction between these variables. This model parameterization retained full information on the crickets since two of the female CHC categories correspond to female species. With the model, we then estimated the probability for courtship success and then the odds ratios for courtship success comparing females with “male-like” CHC profiles to females with other CHC profiles by male species for both conspecific and heterospecific pairs. To control for variability in individual courtship behavior in males across the repeated pairings, we included random intercepts for individual male crickets in the binomial logistic analysis.

We performed time to event analyses (more commonly known as survival analyses) to determine how the rate at which males successfully court a female depends on the species and the female CHC categories. Using the Kaplan-Meier curve, we estimated the probability that a courting event has not yet occurred at every point in time during the courtship trial (0–25 min) for each CHC category, stratifying by male species, and then by pair crossing and whether or not the female has a “male-like” CHC category [26]. Statistical differences between estimated curves were measured by a log-rank test [36]. A Cox proportional-hazards regression model was used to investigate the association of species and the female CHC profiles with the time until courtship success [40, 58]. Using the model, we estimated the hazard ratio for courtship initiation, the ratio of courting rate at any fixed point in time, comparing females with “male-like” CHC profiles to females with other CHC profiles by male species for both conspecific and heterospecific pairings. Similarly, we used a random intercept for individual male crickets to account for variability in individual courtship behavior. We completed the analysis with R 3.6.1 [46].

## Data Availability

The dataset generated and analyzed here is available in the dryad repository under DOI: 10.5061/dryad.cz8w9gj0g

## Abbreviations

CHC: Cuticular hydrocarbon
GCMS: Gas chromatograph mass spectrometry (GCMS)
ML: Male-like females, that is, females that have a typical male CHC profile
